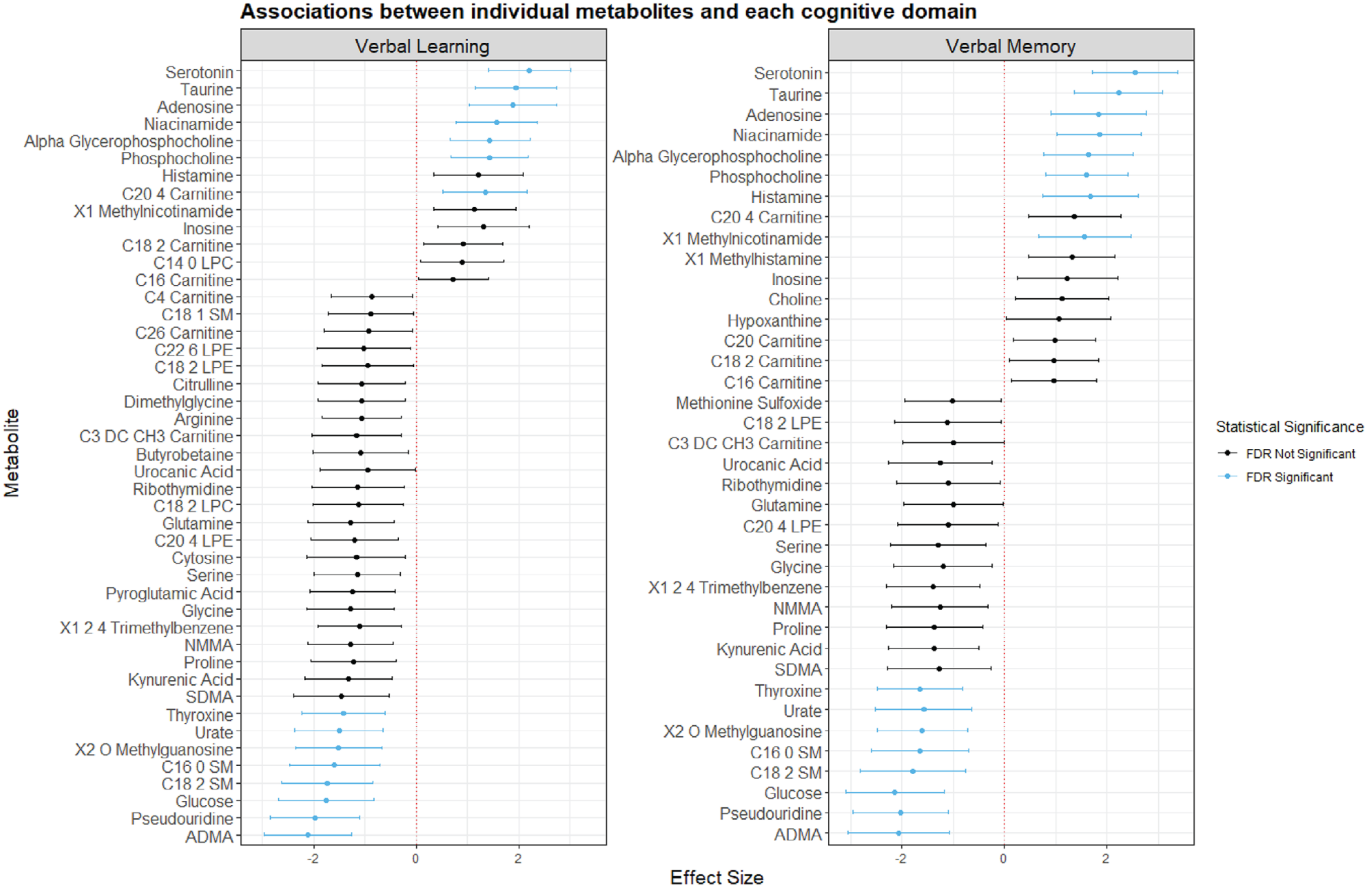# Metabolic changes mediate the link between social determinants and declarative memory in women with and without HIV

**DOI:** 10.1002/alz70856_103135

**Published:** 2025-12-25

**Authors:** Cesar Higgins Tejera, Raha Dastgheyb, Eran Shorer, Qibin Qi, Robbie Burk, Susie Lee, Deborah R Gustafson, Anjali Sharma, Amber D'Souza, Kathleen Weber, Leah H Rubin, Kathryn Fitzgerald

**Affiliations:** ^1^ Johns Hopkins University, Baltimore, MD, USA; ^2^ Albert Einstein College of Medicine, New York City, NY, USA; ^3^ Einstein College of Medicine, Bronx, NY, USA; ^4^ State University of New York Downstate Health Sciences University, Brooklyn, NY, USA; ^5^ Cook County Bureau of Health Services, Chicago, IL, USA; ^6^ Johns Hopkins University, Bethesda, MD, USA

## Abstract

**Background:**

Health determinants such as income and employment significantly influence declarative memory in women with HIV (WWH). These social exposures may contribute to cognitive decline through complex biological mechanisms. We used high‐throughput metabolomics data and causal mediation analysis to identify multiple biological pathways linking adverse socioeconomic conditions and cognitive decline.

**Method:**

A total of 324 women (*n* = 225 WWH) from the New York sites of the Women's Interagency HIV Study completed biennial cognitive testing, starting in 2009 and every two years, which included the Hopkins Verbal Learning Test (HVLT)‐R, a measure of verbal learning and memory. Metabolomic assessment was conducted from serum samples collected prior to or at first cognitive visit using liquid chromatography tandem mass spectrometry. We used multivariable generalized estimating equation models to link peripheral metabolomic profiles and cognitive trajectories in verbal learning and memory. Linear mixed effect models were used to predict subject‐specific trajectories for downstream mediation models. We used regression‐based multiple mediation analyses to estimate the joint effect of multiple metabolic mediators as pathways between income, employment, and cognitive trajectories. We considered a false discovery rate (FDR) of 5% for metabolomic discovery.

**Result:**

Our sample had average age of 43.1 years and were followed‐up for 11.9 years, 63% were non‐Hispanic Black, 76% reported earnings of <24K, and 65% were unemployed. We identified 16 FDR‐corrected metabolites associated with trajectories in verbal memory and 15 with verbal learning. Top metabolites (serotonin, taurine, adenosine, niacinamide, α‐glycerophosphocholine, ADMA, pseudouridine, and sphingomyelins) were common in both domains. Stratified models by serostatus indicated similar results in WWH and women without HIV. In causal mediation analysis, we found that 30% (95%CI: 4%‐100%) of the effect of employment and 18.2% (95%CI: ‐2.1% to 72.3%) of the effect of income on verbal learning was mediated through differences in metabolite levels. We also found that 35.6% (2.7%‐100%) of the effect of employment and 25.4% (95%CI: ‐1.7%‐100%) of the effect of income on verbal memory was mediated through altered metabolomic signatures.

**Conclusion:**

Health determinants influence biological process implicated in cognitive decline. Biosocial interventions are needed to address the conditions that perpetuate poor cognitive health in women.